# Customizing Microfocused Ultrasound With Visualization Treatment for Facial Lifting in Asian Men: Experience and Practical Insights From Korea

**DOI:** 10.1111/jocd.70278

**Published:** 2025-06-09

**Authors:** Je‐Young Park, Wonkyu Hong, Kyou Chae Lee, Hyun Chul Shim, Jeesoo An, Ju Hyuk Park, Hee‐Jin Kim

**Affiliations:** ^1^ Apkoo‐Jung Oracle Dermatology Clinic Seoul Republic of Korea; ^2^ Human Dermatology Clinic Incheon Republic of Korea; ^3^ Hwanggeum Dermatology Clinic Daegu Republic of Korea; ^4^ The Cell Skin Clinic Seoul Republic of Korea; ^5^ Shinsegae Dr. An Dermatology Clinic Seoul Republic of Korea; ^6^ The Heal Dermatology Clinic Seoul Republic of Korea; ^7^ Division in Anatomy and Development Biology, Department of Oral Biology Yonsei University College of Dentistry Seoul Republic of Korea

**Keywords:** Asian men, facial lifting/sculpting, microfocused ultrasound with visualization (MFU‐V), superficial musculoaponeurotic system (SMAS)

## Abstract

**Background:**

Globally, there has been a notable increase in interest and demand for aesthetic treatments among men in recent years. Given the rising trend, it is important to recognize and understand the distinctions in facial anatomy and aesthetic preferences between genders and different ethnicities. When designing treatment for male patients, these differences should be taken into consideration. Microfocused ultrasound with visualization (MFU‐V) is recognized as the current gold standard for skin lifting and tightening. It is the only focused ultrasound device that allows real‐time visualization of the treatment areas, thereby offering personalized and precise delivery of ultrasound energy to target tissue layers. Although guidelines for optimizing MFU‐V treatment have been developed, there is currently no specific guidance for its application in men.

**Aims:**

To provide practical insights for customizing MFU‐V treatments tailored to the unique facial anatomy and aesthetic goals of Asian men.

**Methods:**

Based on the guiding principles in available consensus guidelines and our experience in treating Korean men of different ages presenting with varying concerns, we propose an MFU‐V treatment protocol for facial lifting that takes into account specific facial anatomy characteristics and aesthetic needs of Asian male patients to achieve optimal clinical outcomes.

**Results:**

Relevant examples of the expected outcomes 3 months after applying the protocol are presented.

**Conclusions:**

In our experience, applying this treatment protocol produces excellent skin‐lifting effects, resulting in desired outcomes such as a more defined jawline, improvement in double chin appearance, and slimmer facial appearance.

## 1 Introduction

There is a rising trend of men seeking aesthetic treatments globally [1, 2]. With increased acceptance of aesthetic procedures within society, more men are seeking treatments to enhance their appearance and boost their self‐confidence [3]. Therefore, it is important to be aware of the differences in facial anatomy and aesthetic preferences between genders and different ethnicities [4]. For example, men tend to have 20%–25% thicker skin than women, especially around the forehead, mid‐cheek, jowl, and neck [3]. The Asian face generally has greater mandibular width and a more rounded or square‐shaped face compared with their Western counterparts [5]. In terms of aesthetic ideals, men desire a well‐defined jawline, whereas women pay more attention to skin quality [4]. Asian men prefer a slimmer face compared with Caucasian men, who prefer a stronger and more angular jawline [6]. Gender‐specific facial anatomy and treatment preferences should be taken into consideration when designing aesthetic treatment for male patients [4].

The upward trend in aesthetic treatments is driven primarily by the surge in demand for nonsurgical procedures [1]. The total nonsurgical procedures performed in men rose from 1.4 million procedures in 2020 to 4.1 million in 2021 [1, 2]. This increase in popularity is related to the growing demand for treatment options that have little or no downtime and minimal risk [7]. Various devices using different thermal energy technologies have been developed in response to the rising demand for noninvasive treatment [7]. Intense‐focused ultrasound (IFU) is a non‐invasive, energy‐based technology that delivers ultrasound energy to specific tissue layers, leading to *de novo* collagen production that promotes the lifting and tightening of lax skin [8]. Microfocused ultrasound with visualization (MFU‐V; Ultherapy; Merz North America Inc. Raleigh, N.C., USA) is the only IFU device that offers high‐resolution, real‐time ultrasound imaging of the target area, thus allowing practitioners to visualize and simultaneously treat distinct tissue layers [9]. The thickness of the skin can vary across different areas of an individual's face, leading to differences in the depth of the underlying tissue layers throughout the face [10]. Therefore, real‐time visualization is essential for personalized and precise delivery of microfocused ultrasound energy to the intended treatment depth, which creates thermal coagulation points to stimulate collagen and elastin production, resulting in tissue remodeling, increased viscoelasticity, tissue lifting, and skin thickening and tightening [8, 9].

Several gold standard consensus guidelines for optimizing MFU‐V treatment in Caucasians and Asians have been developed [7, 11, 12]. However, there has not been much discussion about treatment approaches using MFU‐V for men. In this article, we provide practical insights to guide aesthetic practitioners on how to design customized treatment plans to meet the aesthetic needs of Asian men.

## 2 Methods

Based on the framework of the existing gold standard guidelines [7, 11, 12] and our experience in treating Korean men of different ages with varying concerns, we propose a MFU‐V treatment protocol for facial lifting that takes into account the anatomical characteristics and aesthetic preferences of Asian male patients to achieve optimal clinical outcomes. These will guide practitioners to achieve desirable outcomes for male patients and increase patients' satisfaction with their appearance.

### 2.1 Considerations for Aesthetic Treatment in Asian Men

Aesthetic ideals can vary between genders and across different cultures and ethnicities [4, 6]. As such, it is important to consider gender‐specific facial characteristics and aesthetic preferences, as well as cultural and ethnic differences and ideals when planning aesthetic procedures so as to achieve optimal outcomes in Asian men [4, 6].

#### 2.1.1 Facial Anatomy

**FIGURE 1:**
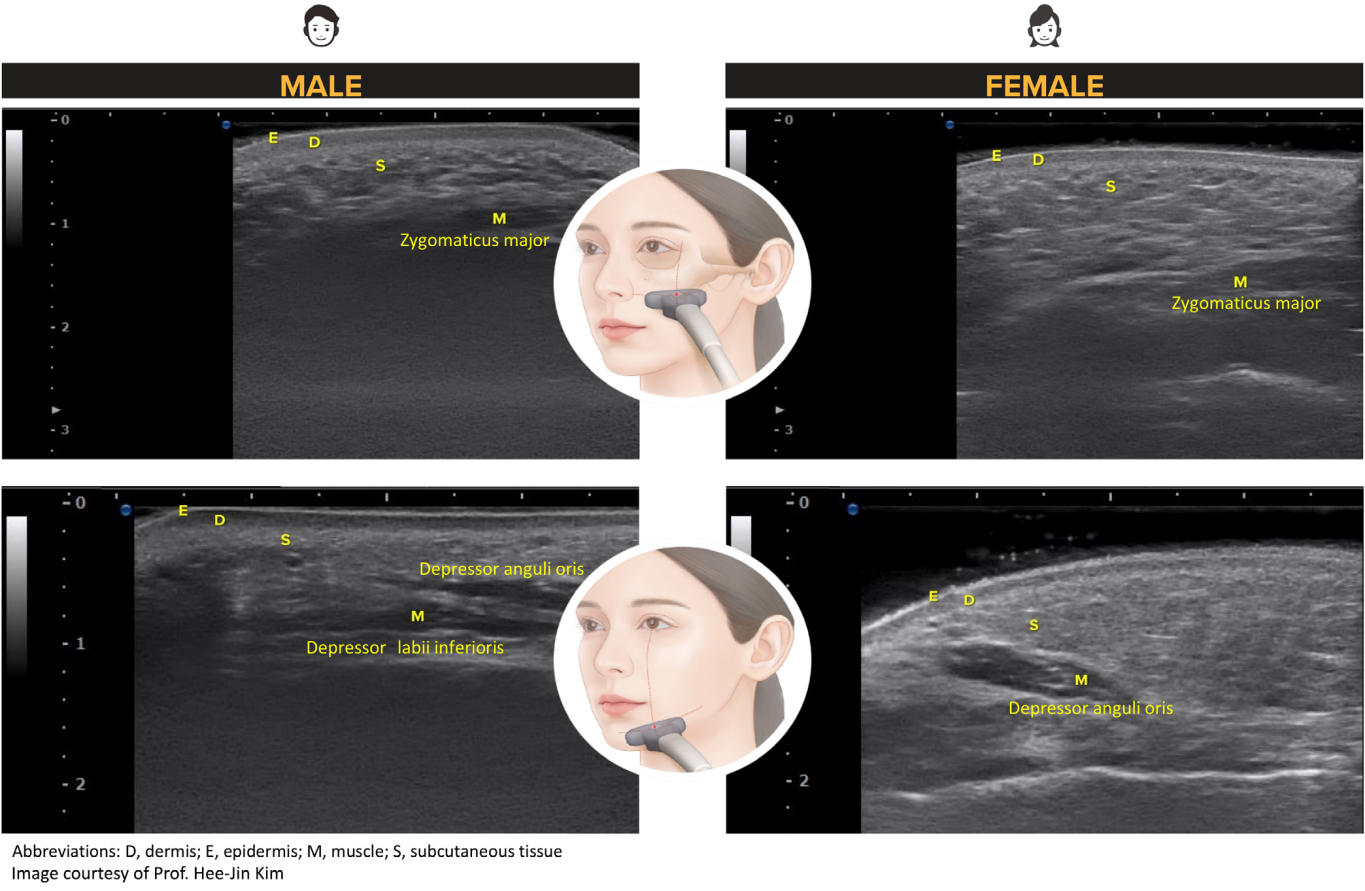
Ultrasound images depicting differences in the thickness of skin layers between male and female patients.

Gender differences in facial characteristics should be noted when planning aesthetic treatment for men [4]. Men tend to have thicker epidermis and dermis but thinner subcutis than women [3] (Figure 1). The distribution of subcutaneous fat across the face also differs between genders, with men having less fat in the cheeks than women [3]. As such, the depth of the underlying tissue layers, including the superficial musculoaponeurotic system (SMAS), at different areas of the face varies across gender [10]. Real‐time visualization is therefore important for treatment customization and precise treatment of the target layers.

#### 2.1.2 Aesthetic Preferences

The cultural ideals and preference for a well‐defined jawline and slimmer face have sparked growing interest in facial sculpting among Asian men [4, 6]. As such, they prioritize jawline contouring and facial lifting over dermal rejuvenation, unlike their female counterparts. There is also a high demand for reducing the appearance of double chin among Asian men [13]. In addition, there is a preference for natural parallel eyebrow lifting instead of arched eyebrows among Asian men.

#### 2.1.3 Treatment Principles

Treatment for Asian men should focus on intensive treatment of the lower face for improvement in double chin appearance and a more defined jawline, and active treatment of the middle face for a slimmer facial appearance through the stimulation of new collagen and lifting of soft tissues. In particular, treatment should focus on stimulating collagen production within deeper layers including the SMAS/superficial fascia and subcutaneous fibrous septa rather than the dermis layer to achieve the desired lifting effect. The SMAS/superficial fascia is a singular, organized fibrous network that connects the facial muscles with the dermis, providing structural support to the face [14]. Given its structural function and involvement in the facial aging process, stimulating the SMAS/superficial fascia can provide more comprehensive lifting than dermal stimulation [10, 15].

### 2.2 Proposed Approach for Optimizing Outcomes of MFU‐V Treatment for Asian Men

Active patient engagement throughout their treatment journey can enhance patient support and improve their overall experience. A detailed aesthetic consultation is crucial to align expectations and treatment goals between the patient and aesthetic practitioners, setting the foundation for a successful treatment. As male patients generally have less experience with aesthetic procedures than their female counterparts, the initial consultation should focus on aligning expectations by providing a detailed and thorough explanation regarding the treatment plans and procedures [4]. It is also crucial to reassure patients that the treatment approach is focused on retaining and/or enhancing their masculine facial features, in line with their personal aesthetic goals. It should be stressed that lifting effects would appear gradually after treatment and touch‐up treatment with other modalities may be required in some instances to achieve the desired outcomes; this is particularly important for patients who have little or no experience with aesthetic treatment or MFU‐V. Detailed explanation should be provided to patients if additional treatments are deemed necessary during follow‐up consultations, as well as the need for maintenance MFU‐V treatment every 12–18 months [7, 11, 12].

**FIGURE 2:**
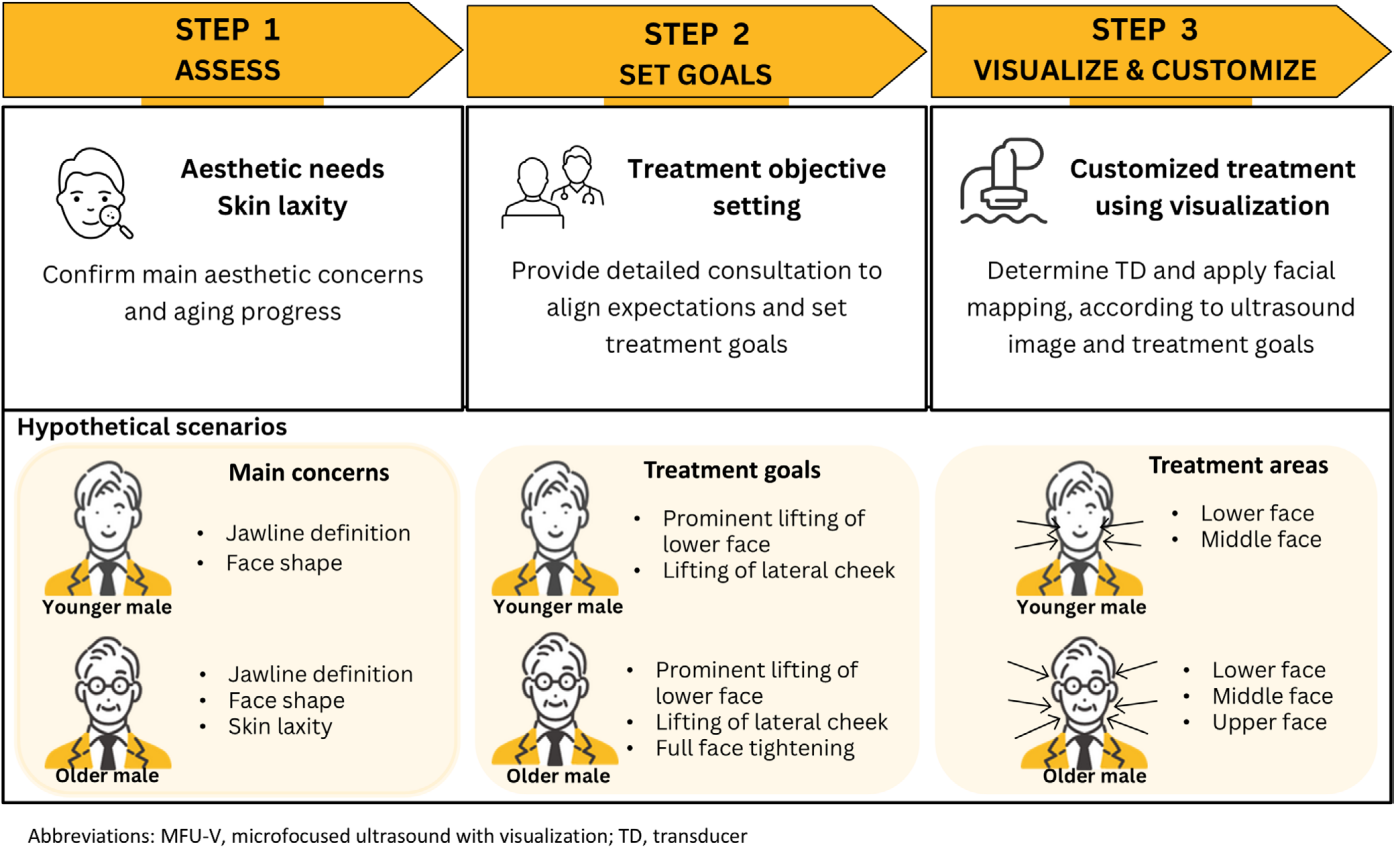
Proposed approach for optimizing outcomes of MFU‐V treatment for Asian men.

Here, we propose a three‐step approach for optimizing outcomes of MFU‐V treatment for Asian male patients (Figure 2).

#### 2.2.1 Step 1: Assess Aesthetic Needs

A thorough assessment of the patient's skin condition is essential prior to treatment. The initial assessment should establish the patient's main aesthetic needs and aging process. For example, men typically present with concerns about jawline definition and face shape, and often express additional concerns of skin laxity as they age.

#### 2.2.2 Step 2: Set Treatment Goals

An open and detailed discussion is crucial to identify areas of concern, align expectations, and set realistic treatment goals. In general, prominent lifting and improvement in the appearance of the double chin in the lower face and lifting of the lateral cheek are desirable in men for a more defined jawline and slimmer face. As the aging process progresses, lifting in the upper face area and full face tightening to improve skin laxity might be desirable in older male patients. However, as the degree of skin aging might differ even for patients of the same age, we recommend customizing treatment according to the individual's aesthetic goals.

#### 2.2.3 Step 3: Customize Treatment Using Visualization

The depth of the tissue layers to be targeted can vary depending on the patient's age, BMI, and treatment area [10]. Therefore, using high‐resolution ultrasound imaging to visualize and identify the target layers within the treatment area is key to customizing treatment and ensuring precise treatment. After identifying the depth of the target layers for the individual patient, practitioners can determine the appropriate treatment parameters needed to achieve the specific aesthetic needs and goals of the patient, including the transducer depth, ultrasound energy level, and number of treatment lines.

#### 2.2.4 Treatment Protocol

**FIGURE 3:**
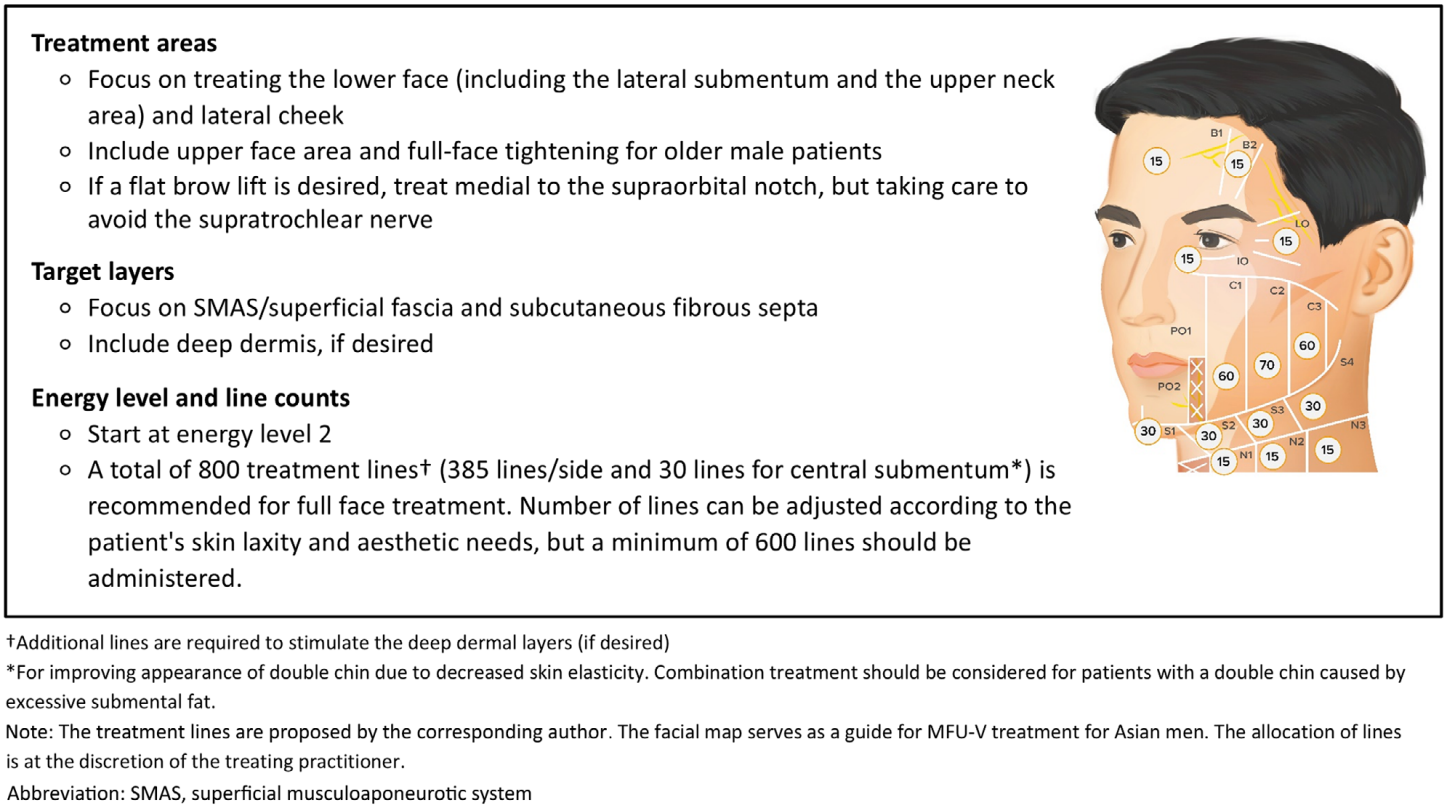
Proposed MFU‐V treatment protocol for facial lifting in Asian men.

Based on our experience in treating Korean men, we developed a MFU‐V treatment protocol for Asian men by integrating gender‐specific facial characteristics and aesthetic preferences into the framework of the existing gold standard guidelines (See figure, Supporting Information S1 that illustrates the MFU‐V 4.5 mm treatment guidelines targeting the SMAS/superficial fascia for women) [7, 11, 12]. Figure 3 illustrates the proposed MFU‐V treatment protocol for facial lifting in Asian men. The treatment areas, target layers, and actual number of treatment lines should be adjusted according to the facial characteristics and aesthetic needs of the individual male patient. In general, more treatment lines are directed to the lower face and middle face, and deeper layers are targeted to focus on jawline contouring and facial lifting rather than dermal rejuvenation for Asian men.

Treatment for Asian men should focus on intensive treatment of the lower face, including the lateral submentum and upper neck to produce a tightening effect on the platysma layer along the mandible, thereby improving the appearance of double chin and achieving prominent lifting for a more defined jawline. The lateral cheek should be actively treated for a slimmer facial appearance through the stimulation of new collagen and lifting of soft tissues. With aging, lifting in the upper face area and full‐face tightening to improve overall skin laxity are also desirable. Treatment for Asian men includes the central forehead area in addition to the lateral brow area. For a flat brow lift, treatment is directed medial to the supraorbital notch, taking care to avoid the supratrochlear nerve.

In terms of the target layers, we recommend focusing on deeper layers including the SMAS/superficial fascia and subcutaneous fibrous septa to provide more comprehensive lifting effects. We recommend starting at energy level 2 and delivering a minimum of 600 lines up to a total of 800 lines for full face and upper neck treatment. Additional lines can also be delivered to the deep dermis as desired for more superficial skin quality improvements.

Other treatment modalities can be combined with the MFU‐V protocol to achieve the desired outcomes when necessary. The selection of additional treatment modalities should be based on the individual patient's aesthetic concerns and facial anatomy, as well as the unique properties of available treatment modalities. For instance, combined treatment with botulinum neurotoxin A (BoNT‐A) should be considered for men with prominent masseter and/or platysma muscle or hypertrophic parotid gland to reduce the width and shape of the lower face and jawline [16]. As masseter and parotid gland reduction usually requires high doses of BoNT‐A, incobotulinumtoxinA (INCO, Bocouture/Xeomin; Merz Pharmaceuticals GmbH, Frankfurt, Germany) is a rational option as it is highly purified and free of unnecessary components such as complexing proteins, thereby reducing the risk of immunoresistance and secondary nonresponse [17, 18]. For men with excessive submental fullness, combination treatment with deoxycholic acid should be considered to reduce submental fat and improve the overall jawline appearance [19, 20, 21].

### 2.3 Practical Tips for Applying the Protocol for Optimal Outcomes

We propose several practical tips for applying the MFU‐V treatment protocol to Asian men, including the ideal candidate patients, approach to comfort management, special considerations for the treatment areas, and appropriate maintenance interval. The details are summarized in Table 1.

**TABLE 1:** Practical tips for applying the MFU‐V treatment protocol for optimal outcomes.

Ideal candidate patients	○ Patients in their 30s to 50s with a less defined jawline ○ Patients with mild‐to‐moderate skin laxity ○ Mild eyebrow ptosis ○ Patients who are reluctant to receive invasive procedures ○ Patients who desire treatment with minimal downtime ○ Patients who desire gradual and continued improvement in treatment effects
Considerations for the treatment areas	○ Prior to treating the submentum and upper neck area, it is essential to carefully observe patients with ample hair or thick beard around the jaw area, as there could be cuts that can cause irritations in the shaved area. Special attention should be given to monitoring for folliculitis ○ When treating the central forehead area, avoid nerve distribution and treat medially to the frontal notch
Pain management	○ Pain management strategy should follow the gold standard guidelines [7, 11, 12] ○ Consider an individualized pain regimen
Interval for maintenance treatment	○ A maintenance treatment session is recommended once every 12–18 months as per the gold standard guidelines recommendations [7, 11, 12] ○ Practitioners may consider treating every 12 months to maintain the optimal lifting effect

Abbreviation: MFU‐V, microfocused ultrasound with visualization.

In addition to setting appropriate expectations in male patients prior to MFU‐V treatment, careful selection of candidate patients can also lead to higher patient satisfaction. Patient comfort during and after MFU‐V treatment is an important aspect that should not be overlooked as it can improve patient satisfaction and adherence to future treatment [22]. As patient comfort varies depending on their pain tolerance and treatment areas, practitioners should consider using a stepwise individualized approach for pain management [7, 11, 12]. Based on individual patient's pain tolerance and contraindications to pain medications, physicians can tailor their strategies to use different medications such as topical analgesics and/or oral analgesics, and distraction techniques such as vibratory devices or forced air cooling before or during treatment for comfort management [7, 11, 12]. Patients are recommended to receive maintenance treatment every 12–18 months, as per current recommendations in available gold standard guidelines [7, 11, 12]. Practitioners may consider treating every 12 months for patients who wish to maintain the optimal lifting effect.

## 3 Results

### 3.1 Clinical Cases Illustrating the Treatment Outcomes

**FIGURE 4:**
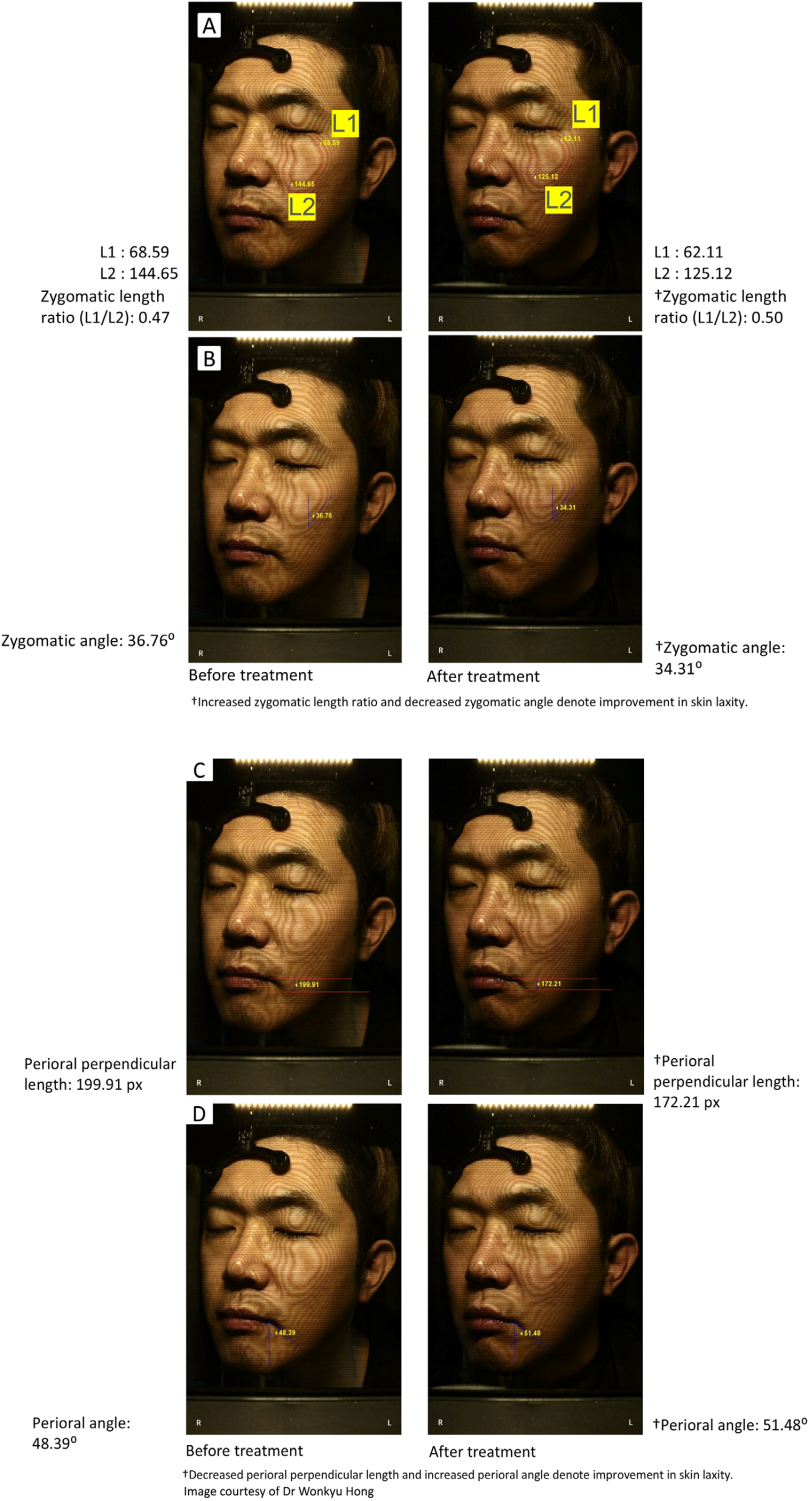
Lifting effects observed in the middle (A and B) and lower face (C and D) 12 weeks after a single treatment of MFU‐V, as assessed by ImageJ.

**FIGURE 5:**
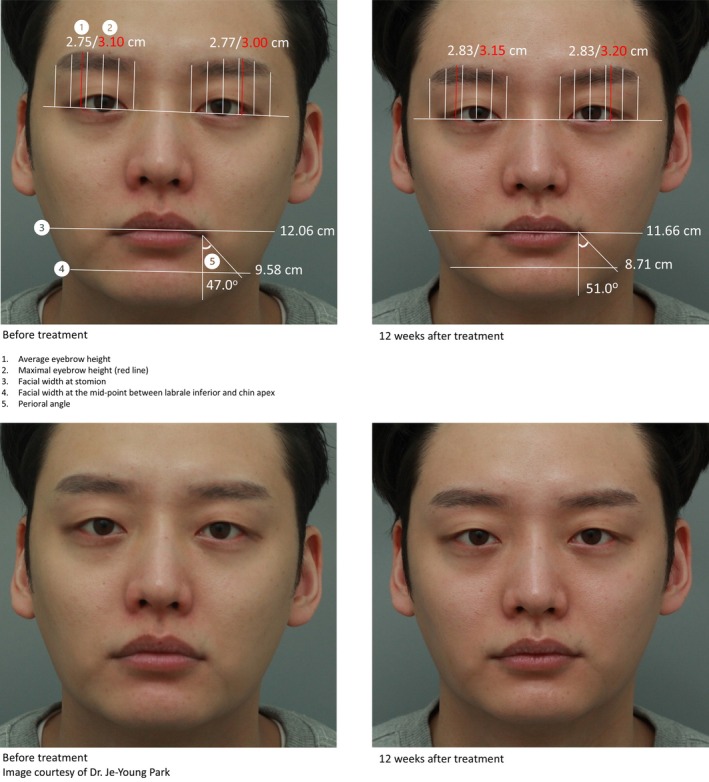
Naturally lifted eyebrows and middle face, narrowed lower face, and mouth angle elevation observed at Week 12 after MFU‐V treatment, as assessed using ImageJ. Bottom row shows photos without annotation.

We have collectively treated 2000 male patients using the proposed treatment protocol, with the accompanying Figures 4 and 5 showing examples of the expected outcomes 3 months after applying the protocol.

A 43‐year‐old man presented with concerns of mild‐to‐moderate skin laxity and an undefined jawline due to aging. After discussing treatment goals and reviewing the patient's facial anatomy with real‐time ultrasound imaging, a total of 715 lines at energy level 2 was applied to the middle and lower face including the submentum, targeting the SMAS/superficial fascia (335 lines) and subcutaneous fibrous septa (380 lines) using a 4.5 mm transducer and a 3.0 mm transducer, respectively. Standardized photographs were taken before treatment and 12 weeks after treatment using F‐RAY (BEYOUNG Ltd., Seoul, Korea), an imaging device based on moiré 3D technology to create topographic maps [23]. The following parameters: the zygomatic length, zygomatic angle, perioral perpendicular length, and perioral angle were measured using ImageJ [21]. The definitions of these parameters have been previously described [23] (see Document, Supporting Information S2, that describes the definition for each parameter). Figure 4 depicts lifting effects in the middle and lower face 12 weeks after a single treatment of MFU‐V, as assessed by ImageJ. The zygomatic length ratio increased from 0.47 at baseline to 0.50 at Week 12 (Figure 4A). The zygomatic angle decreased from 36.76° at baseline to 34.31° at Week 12 (Figure 4B). Together, the improvements in zygomatic length ratio and zygomatic angle indicate substantial lifting effects in the middle face, which contributed to a slimmer facial appearance. The perioral perpendicular length decreased from 199.91 px at baseline to 172.21 px at Week 12 (Figure 4C). In addition, the perioral angle increased from 48.39° at baseline to 51.48° at Week 12 (Figure 4D). Assessments of both parameters revealed lifting in the lower face area after 12 weeks of MFU‐V treatment, resulting in a more prominent jawline.

A 32‐year‐old man presented with concerns of mild eyebrow ptosis, blunted jawline, and widening facial shape. After discussing treatment goals and reviewing the treatment areas using real‐time ultrasound imaging, a total of 800 lines at energy level 2 was applied to the central forehead, lateral eyebrow, lateral and infraorbital area, anterior and posterior cheek, submentum, and upper neck, targeting the SMAS/superficial fascia (560 lines) and subcutaneous fibrous septa (240 lines) using a 4.5 mm transducer and a 3.0 mm transducer, respectively. Photographs of front views were taken before treatment and 12 weeks after treatment. Eyebrow heights, facial widths at stomion and at the mid‐point between labrale inferior and chin apex, and perioral angle were measured using ImageJ [24] (see Document, Supporting Information S3, that describes eyebrow height measurement and calculations). Figure 5 illustrates naturally lifted eyebrows and middle face, narrowed lower face, and mouth angle elevation observed at Week 12 after MFU‐V treatment, as assessed by ImageJ. The average eyebrow height (AEH) on the right and left sides increased from 2.75 cm and 2.77 cm, respectively, at baseline to 2.83 cm for each side at Week 12. Similarly, the maximal eyebrow height (MEH) on the right and left sides also increased from 3.10 cm and 3.00 cm at baseline to 3.15 cm and 3.20 cm at Week 12, respectively. Facial widths at stomion and at the mid‐point between labrale inferior and chin apex decreased from baseline at Week 12. In addition, the perioral angle increased from 47.0° at baseline to 51.0° at Week 12, denoting improvement in skin laxity around the area. Together, these measurements showed lifting effects pan‐facially, resulting in a rejuvenated upper face and slimmer lower face with a more defined jawline.

We recognize several factors that can affect the evaluation of treatment outcomes, such as standardizing image capture or image analysis settings. To improve consistency, we suggest applying a standardized scoring system like SEPIA [25] to ensure that the quality of before and after photographs is uniform, as well as recording and using the same image capture or analysis settings for the same patient.

## 4 Conclusions

This article describes a unique approach to designing a personalized MFU‐V treatment, drawing from the guiding principles in available consensus guidelines [7, 11, 12] and our experience treating Korean men across different ages with varying aesthetic concerns. The treatment protocol is tailored to address specific facial anatomy characteristics and aesthetic needs of Asian male patients, aiming to achieve optimal clinical results. Based on our experience, applying this treatment protocol produces excellent skin‐lifting effects, leading to outcomes such as a more defined jawline, improvement in double chin appearance, and slimmer facial appearance. Recognizing that facial features and treatment preferences may differ in other populations, we recommend practitioners use this guide as a starting point and modify the protocol to suit their patient's needs. Since male patients generally have little or no experience with aesthetic procedures compared with their female counterparts, it is especially critical for practitioners to provide detailed explanations regarding the treatment plan and align expectations and treatment goals with their male clients, including touch‐up treatment with other modalities that may be required in some instances to achieve the desired outcomes.

## Author Contributions

All authors provided clinical expertise and critical insights for treatment customization. They contributed to the conceptualization and development of the article and were involved in critically revising it for important intellectual content. They approved the final version and agreed to be accountable for all aspects of the work.

## Disclosure

The authors have nothing to report.

## Ethics Statement

The authors have nothing to report.

## Consent

All of the patients whose photographs are used in this article provided written informed consent for the publication of their photographs.

## Conflicts of Interest

The authors declare no conflicts of interest.

## Supporting information


**Supporting Information S1.** Figure that illustrates the MFU‐V 4.5 mm treatment guidelines for women.

**Supporting Information S2.** Document that describes the definition for each parameter.

**Supporting Information S3.** Document that describes eyebrow height measurement and calculations.

## Data Availability

Data sharing is not applicable to this article as no datasets were generated or analyzed.
