# Supplementary figures and images for: Customizing Microfocused Ultrasound With Visualization Treatment for Facial Lifting in Asian Men: Experience and Practical Insights From Korea
Source: J Cosmet Dermatol. 2025 Jun 9;24(6):e70278. doi: 10.1111/jocd.70278 (PMC12147194; doi:10.1111/jocd.70278)

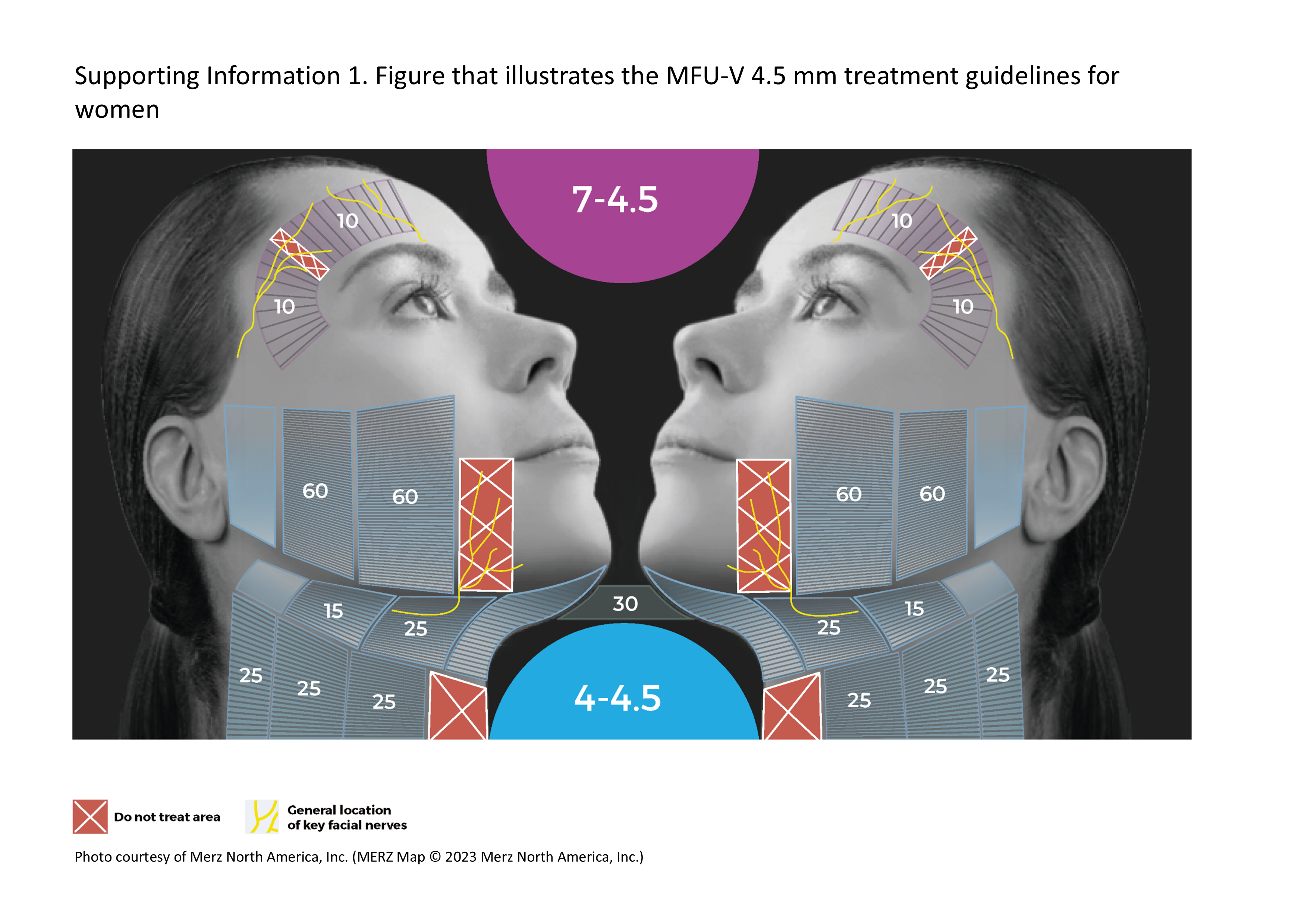

Supplement: Supplementary file 1 — Supporting Information S1. Figure that illustrates the MFU‐V 4.5 mm treatment guidelines for women. [file JOCD-24-e70278-s001.png]
