# Supplementary material for: Customizing Microfocused Ultrasound With Visualization Treatment for Facial Lifting in Asian Men: Experience and Practical Insights From Korea
Source: J Cosmet Dermatol. 2025 Jun 9;24(6):e70278. doi: 10.1111/jocd.70278 (PMC12147194; doi:10.1111/jocd.70278)
Supplement: Supplementary file 2 — Supporting Information S2. Document that describes the definition for each parameter. [file JOCD-24-e70278-s002.docx]

# Supporting information 2. Document that describes the definition for each parameter.

- The zygomatic length is the distance (L) between the first and second adjacent circles at the cheek area (**Figure 4**).
- The minimum length (L1) and maximum length (L2) were used to calculate the zygomatic length ratio (L1/L2); increased ratio denotes improvement in skin laxity.
- The zygomatic angle is the distance between the straight line drawn vertically from the center of the concentric circle of the cheek and the tangent line drawn from the third circle of the cheek; decreased angle denotes improvement in skin laxity.
- The perioral perpendicular length is the distance between the horizontal line drawn at the oral commissure and the second moiré curve; decreased length denotes improvement in skin laxity.
- The perioral angle is the angle between the perpendicular line from the oral commissure and the specific tangent line of the curve connecting the commissure; increased angle denotes improvement in skin laxity.
