# Supplementary material for: Customizing Microfocused Ultrasound With Visualization Treatment for Facial Lifting in Asian Men: Experience and Practical Insights From Korea
Source: J Cosmet Dermatol. 2025 Jun 9;24(6):e70278. doi: 10.1111/jocd.70278 (PMC12147194; doi:10.1111/jocd.70278)
Supplement: Supplementary file 3 — Supporting Information S3. Document that describes eyebrow height measurement and calculations. [file JOCD-24-e70278-s003.docx]

# Supporting information 3. Document that describes eyebrow height measurement and calculations.

- Eyebrow heights were measured from the medial canthi connection line to the top of the eyebrow at each point (**Figure 5**). Six measurements were taken by moving horizontally from the medial end of the eyebrow to the lateral end at equal intervals.
- The average of the measured eyebrow heights was calculated and reported as the average eyebrow height (AEH).
- The maximal eyebrow height (MEH) is the maximum distance from the medial canthi connection line to the eyebrow.
